# Revisiting the Classification of Physisorption Isotherms with Classical Density Functional Theory

**DOI:** 10.1021/acs.jpcb.5c08129

**Published:** 2026-02-02

**Authors:** Thomas Bernet, Corentin Canu, George Jackson

**Affiliations:** † Department of Chemical Engineering, Sargent Centre for Process Systems Engineering, 4615 Imperial College London, South Kensington Campus, London SW7 2AZ, U.K.; ‡ Collège des Sciences et Technologies pour l’Energie et l’Environnement, Université de Pau et des Pays de l’Adour, Anglet 64600, France

## Abstract

Understanding the mechanisms of gas adsorption/desorption and liquid intrusion constitutes a fundamental field that spans physics, chemistry, and engineering and leads to applied problems, such as the characterization of porous materials and porosimetry. The physisorption of gases is typically analyzed at constant temperature for various pressures, and the corresponding isotherms have been classified in standard IUPAC reports, mainly based on empirical considerations. We use classical density functional theory (DFT) to predict the microscopic structure of confined fluids and determine physisorption isotherms for gases, liquids, and supercritical fluids by considering a large range of thermodynamic conditions. The effects of temperature, pore size, and intensity of the fluid–solid interactions are systematically studied. New types of isotherms are identified, and we reinterpret several mechanisms of adsorption and desorption. We propose a new classification of physisorption isotherms organized from a fundamental perspective based on thermodynamic considerations.

## Graphical abstract

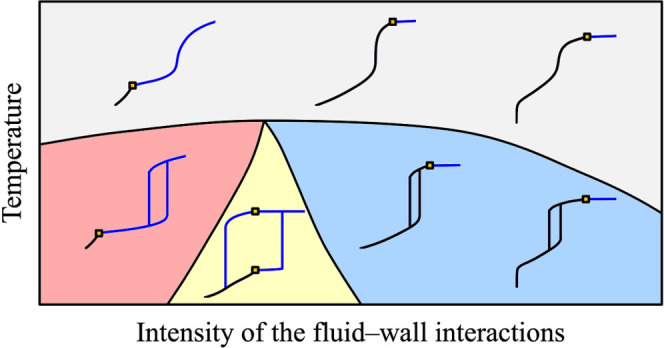

## 1 Introduction

The characterization of porous materials relies on a wide range of techniques, including gas adsorption and liquid intrusion, to determine features such as surface area, pore size distribution, and porosity.^1,2^ Gas adsorption typically involves an increase in the amount of molecules of the gas on the surface of a solid substrate due to attractive gas–solid interactions. These interactions depend on the nature of the material, e.g., zeolites, metal–organic frameworks (MOFs), activated carbons, or silica gels, and can correspond to physisorption (driven by van der Waals forces) or chemisorption (when chemical reactions on the surface of the material are involved). Standard techniques and experimental procedures have been developed to study the mechanisms of gas adsorption, e.g., using nitrogen at 77 K or argon at 87 K, but can also be applied at higher temperatures to supercritical fluids. When the fluid–solid interactions are strong enough, the fluid confined into the pore can condense, such that the pore is saturated (i.e., filled), while the bulk fluid out of the pore is still in a gas state. By contrast, when the fluid–solid interactions are very weak or repulsive, the fluid confined into the pore can be a gas, while the bulk fluid is in a liquid state. Such a feature is used with nonwetting liquids, e.g., for techniques of porosimetry based on mercury intrusion.

Porous materials can be characterized by analyzing the shape of the physisorption isotherms, using gas adsorption or liquid intrusion, and by expressing the amount adsorbed as a function of the bulk pressure. The adsorption and desorption branches of an isotherm are obtained by considering, respectively, an increase and a decrease of the bulk pressure *P* (typically for *P* < *P* ^0^ for gas adsorption and from *P* ^0^ for liquid intrusion, where *P* ^0^ is the vapor (saturation) pressure of the bulk fluid). It is necessary to consider both adsorption and desorption branches as the isotherms can be irreversible and exhibit hysteresis. The shape and the possible irreversibility of an isotherm depend on many physical properties, such as temperature, pore size, and geometry, and the intensity of the fluid–solid interactions, which can all be used for the characterization of porous substrates. In particular, gas adsorption can be applied to the characterization of micropores (i.e., for pore sizes smaller than 2 nm), mesopores (i.e., for pore sizes between 2 and 50 nm), and macropores (i.e., for pore sizes larger than 50 nm), while mercury intrusion is commonly applied to wide mesopores and macropores.^1,3^

Many theoretical methods and empirical equations have been developed to study the mechanisms of physisorption and analyze the corresponding isotherms.^2^ Langmuir established an equation^4^ for monolayer gas adsorption, and many extensions have been developed for more complex mechanisms, e.g., with multilayer adsorption and irreversible isotherms.^2,5,6^ Theoretical and experimental techniques of characterization have become specialized to gas adsorption (e.g., nitrogen porosimetry) or liquid intrusion (e.g., mercury porosimetry), treating gas–solid and liquid–solid interfaces with different approaches. Classifications of physisorption isotherms have been developed over decades^1,7–11^ to enable the standardization of techniques for gas adsorption and highlight the relations between the shapes of isotherms (and the shapes of hysteresis cycles^1,12^ ) and the corresponding structures of porous materials. The main classification for gas physisorption was published by Thommes et al. in 2015 as an IUPAC report.^1^ However, the current methods introduce a systematic distinction between gases and liquids, and as a consequence, none of the usual classifications can be applied to the isotherms obtained by liquid intrusion. The absence of a unique framework might be a limitation to a better understanding of both adsorption and intrusion mechanisms in relation to the standard classifications of physisorption isotherms. Such a limitation can be overcome by taking the vapor–liquid equilibrium (VLE) of the bulk fluid into account in the approach, as is typically done from a physical and thermodynamic perspective.

In our current work, we revisit adsorption–desorption and intrusion mechanisms for a fluid confined within a porous material in contact with a bulk fluid that can be a gas, a liquid, or a supercritical fluid. The VLE of the bulk fluid is systematically considered in the study as well as the state of the confined fluid until the saturation of the pore. Our approach implies that the classification of physisorption isotherms must be extended and must rely on a more fundamental basis. Such a goal can be achieved by using approaches based on statistical physics instead of specific empirical equations. Molecular simulation could be an option; however, it would take a long computational time to get enough points for each isotherm and explore a wide range of thermodynamic conditions. Determining the stable state of both the bulk and the confined fluids can constitute another difficulty, particularly when hysteresis cycles are present in the isotherms. To overcome these issues, we use an approach based on the statistical associating fluid theory^13–15^ (SAFT) combined with classical density functional theory^16^ (classical DFT). On one hand, SAFT is a molecular equation of state (EoS) that can be used to determine the thermophysical properties of real fluids, e.g., for a bulk fluid with homogeneous density. We choose the SAFT-VR Mie version^17^ of SAFT, which is equivalent for spherical non-associating fluids to the more recent SAFT-γ Mie group-contribution version^18–20^ (used in many studies and modern applications^21–25^ ). On the other hand, classical DFT can be used to determine the microscopic structure of inhomogeneous fluids, like confined fluids.^16^ The thermodynamic properties at equilibrium of the confined fluid, including the surface tension and amount adsorbed, can be derived from the microscopic structure. The SAFT and classical DFT frameworks can be combined^26–31^ as they both rely on the same molecular modeling to determine the effects of confinement on real fluids. Our recent SAFT–DFT approach^29^ can be used to consider gases and liquids within the same framework as the VLE of the bulk fluid can be determined from the EoS and the possible hysteresis cycle of the physisorption isotherm can be obtained with classical DFT.

We summarize the various aspects of our study with the following questions:

(1) For the physisorption of gases, can we obtain all of the types of isotherms of the 2015 IUPAC classification^1^ with classical DFT, and can we find new types of isotherms? (2) What possible topologies and new types of physisorption isotherms are observed by considering gases, liquids, and supercritical fluids within the same theoretical framework? (3) Can we revisit the mechanisms of adsorption/desorption and intrusion into macropores, and how do these mechanisms affect the reversibility of the isotherms? (4) What are the topologies of the isotherms of physisorption for fluids confined into micropores? (5) What is the effect of the temperature on the shape of the physisorption isotherms?

The methodology employed in our work can be found in Section 2. We present the existing classifications of physisorption isotherms (Section 2.1), the DFT framework used (Section 2.2), and the possible topologies of the isotherms (Section 2.3). Results are listed in Section 3. We detail cases for macropores (Section 3.1), micropores (Section 3.2), and the effect of temperature (Section 3.3), and we summarize our results in a new classification of physisorption isotherms (Section 3.4). Concluding remarks are provided in Section 4.

## 2 Methods

### 2.1 Classifications of Physisorption Isotherms

The current IUPAC classification of physisorption isotherms was introduced by Thommes et al. in 2015.^1^ It was an update of the former IUPAC classification established by Sing et al. in 1985,^9^ which was itself based on the classification of Brunauer, Deming, Deming, and Teller (BDDT)^7^ of 1940. We refer to these three classifications as IUPAC 2015, IUPAC 1985, and BDDT, respectively. The isotherms are represented in terms of the amount of fluid adsorbed as a function of the relative pressure *P*/*P* ^0^, where *P* ^0^ is the vapor pressure of the bulk. For the adsorption of gases, six main types of isotherms have been identified empirically, denoted as Types I–VI. They are shown in Figure 1 (with their possible subtypes).

**Figure 1:**
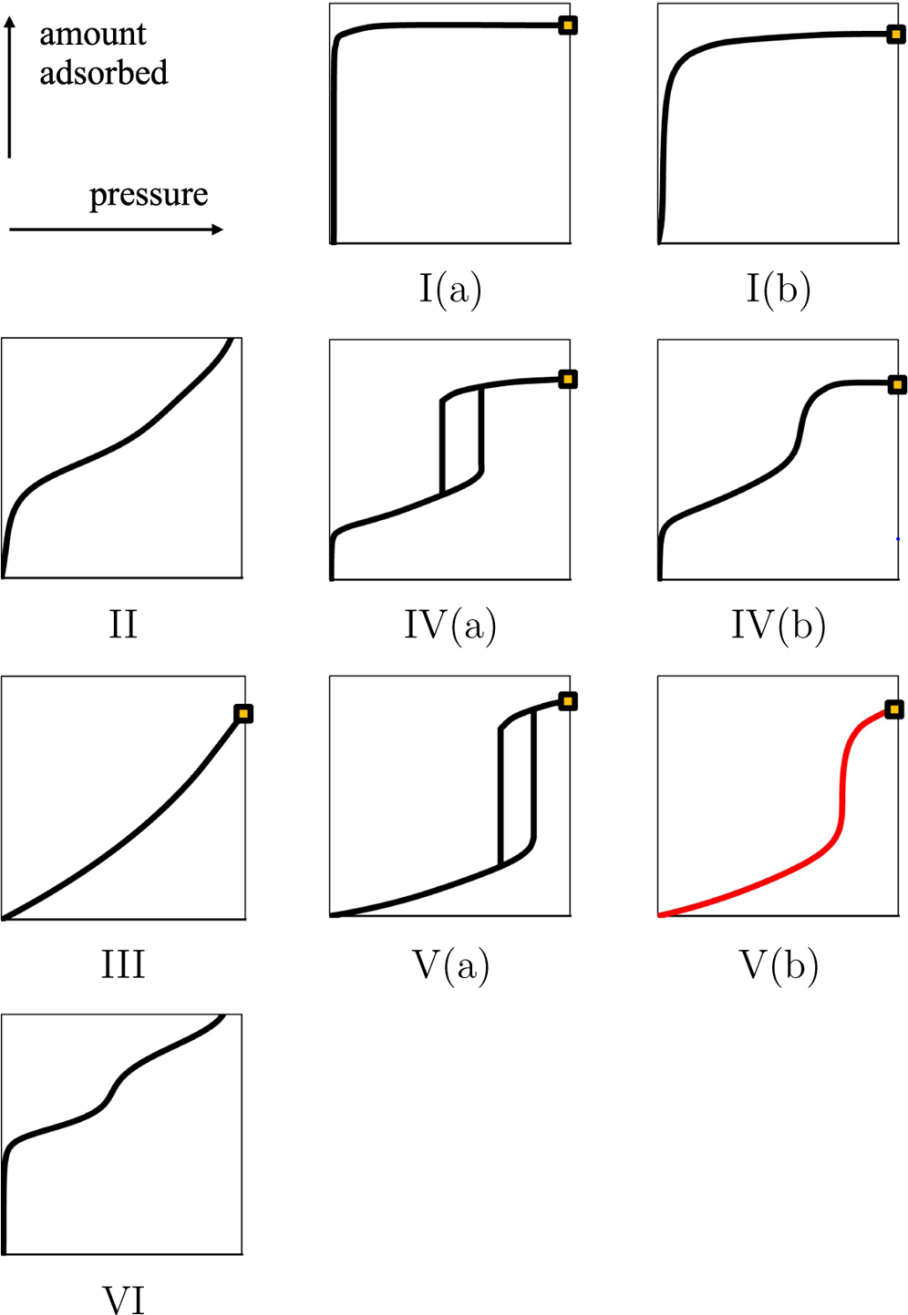
Classification of physisorption isotherms for gases. We rename Type V isotherms of the IUPAC 2015 classification^1^ as Type V­(a) and add Type V­(b) (in red) by analogy with Types IV­(a) and IV­(b). The gold squares indicate the saturation of the bulk fluid at vapor pressure *P* ^0^.

Isotherms of **Type I** have a concave shape. They can be found for micropores (i.e., for pore size *H* < 2 nm). The sharp increase of the amount adsorbed seen at low pressures occurs because of the formation of the first layer of adsorption in the porous material, which can be the only possible layer for the smallest pores. At higher pressures, the amount adsorbed does not vary significantly, which indicates that the pore is saturated for *P* < *P* ^0^. Isotherms of Type I can be described by the equation of Langmuir^4^ and its extensions^2^ (derived from kinetics or thermodynamics considerations). Type I can be found in all the standard classifications of physisorption isotherms but was split in IUPAC 2015 into **Type I­(a)** for small micropores (up to 1 nm) and **Type I­(b)** for wider micropores.

**Type II** of BDDT, IUPAC 1985, and IUPAC 2015 can be observed for macropores (i.e., for *H* > 50 nm) and non-porous materials. It has a sigmoid shape, which points out the formation of the first layers at low pressure due to strong fluid–wall interactions and the formation of additional layers at higher pressure. The saturation of the pore is not reached for *P* < *P* ^0^, and the amount adsorbed diverges at *P* ^0^ for nonporous surfaces, which corresponds to the formation of a liquid film of any possible thickness. The mechanism of adsorption involving multiple layers adsorbed at a single surface was proposed by Brunauer, Emett, and Teller in 1938 and is referred to as the BET theory.^5^

Isotherms of **Type III** have a convex shape, which occurs when there is no formation of the first layer of adsorption at low pressure due to weak fluid–wall interactions. Type III is typically found for macropores, and the isotherms can be modeled with the BET theory.^5^ As with Type II isotherms, the adsorbed amount can diverge at *P* ^0^ for non-porous materials and the largest macropores, when the effects of confinement can be neglected, as can be seen in the BDDT and IUPAC 1985 classifications. By considering adsorption in mesopores with classical DFT, Balbuena and Gubbins (1993)^10^ found isotherms that have a convex shape (like Type III in BDDT and IUPAC 1985) but a finite amount adsorbed at *P* ^0^ (contrary to Type III in BDDT and IUPAC 1985), while the saturation is not reached for *P* < *P* ^0^. They referred to these isotherms as Type VII and mentioned that they are related to the phenomenon of capillary evaporation.^10^ In IUPAC 2015, the definition of Type III is generalized to any convex isotherm with or without divergence at *P* ^0^, which includes the proposed Type VII of Balbuena and Gubbins.

The **Type IV** family represents physisorption isotherms that are similar to Type II at low pressure (which indicates the formation of the first layers of adsorption because of strong fluid–wall interactions) but that reach saturation at higher pressures (which indicates that the pore is filled), typically for mesopores (i.e., for 2 nm ≤ *H* ≤ 50 nm). Most of the isotherms of Type IV found in the literature are not reversible, such that the adsorption and desorption branches of the full isotherm exhibit a hysteresis cycle for *P* < *P* ^0^. It is the signature of capillary condensation, which indicates that the confined fluid is subject to a phase transition, while the bulk fluid remains in a gaseous state. The mechanisms of adsorption and desorption can be described with the Barrett, Joyner, and Halenda (BJH) theory[Bibr ref6] (1951), based on the Kelvin equation and kinetics considerations. The hysteresis cycle was not described in BDDT as only the adsorption branch of the isotherm was taken into account, and the irreversibility and the hysteresis cycle of Type IV were introduced and discussed in IUPAC 1985. Two subtypes have been distinguished in the modern classification of IUPAC 2015: **Type IV­(a)**, which is irreversible and exhibits a hysteresis cycle, and **Type IV­(b)**, which is reversible and does not exhibit a hysteresis cycle. Type IV­(b) follows a concave–convex–concave shape on increasing the bulk pressure: the increase of the amount adsorbed at low pressure still corresponds to the formation of the first layer, and the increase at higher pressure corresponds to the filling of the pore until saturation but without a phase transition (i.e., the confined fluid behaves like a supercritical fluid, while there is VLE for the bulk fluid). As mentioned in IUPAC 2015, Type IV­(b) can be found for small mesopores (of conical and cylindrical geometries). However, the role of the temperature must be clarified. By analogy with the behavior of a bulk fluid that becomes supercritical for *T* > *T*_c_, Type IV­(b) isotherms should be found with any Type IV­(a) systems by increasing the temperature. A critical temperature *T*_c_^conf^ can be found for the confined fluid and must be lower than the critical temperature *T*_c_ of the bulk fluid because of the presence of the fluid–wall interactions. The value of *T*_c_^conf^ depends on the properties of both fluid and wall, including the pore size *H*, pore geometry and roughness, and the intensity of the fluid–wall interactions ε_fw_.

**Type V** is to Type III what Type IV is to Type II. There is no formation of the first layer at low pressure, and the pore is filled until saturation at higher pressure for *P* < *P* ^0^, i.e., Type V can be found for mesopores with weak fluid–wall interactions and can be described by the BJH theory.^6^ Only the adsorption branch is shown in BDDT. In IUPAC 1985 and IUPAC 2015, both the adsorption and desorption branches are shown, constituting a hysteresis cycle. In contrast with Type IV isotherms, no subtypes have been defined in IUPAC 2015, and Type V is systematically represented with a hysteresis cycle; however, we feel that it is important to acknowledge this behavior. In our current work, we rename Type V isotherms as **Type V­(a)** for irreversible isotherms, and we define a possible **Type V­(b)** when the isotherms of Type V are reversible, as can be seen in Figure 1. The absence of Type V­(b) in modern classifications could be justified because of the uncommon or difficult conditions of observation rather than physical considerations. The separate effects of pore size and temperature must be clarified to explain the transition from Type V­(a) to Type V­(b), by analogy with the transition from Type IV­(a) to Type IV­(b).

**Type VI** isotherms are characterized by multiple and successive layers, observed for nonporous materials with smooth walls at low temperature. No hysteresis cycle is mentioned, as for Types II and III. Type VI was absent from BDDT but included in IUPAC 1985 and IUPAC 2015.

The IUPAC 2015 classification for gases can be extended to supercritical fluids when the temperature *T* is higher than the critical temperature *T*_c_ (by considering the pressure *P* instead of the relative pressure *P*/*P* ^0^). However, several types of isotherms of IUPAC 2015 cannot exist for supercritical fluids, like Types IV­(a) and V­(a), because of the absence of phase transition for *T* > *T*_c_ for both bulk and confined fluids.

Donohue and Aranovich^11,32^ analyzed the behavior of isotherms from Type I to Type V, using a lattice model of adsorption for gases and supercritical fluids, from temperatures close to the freezing point to temperatures above *T*_c_. Both Types IV­(a) and IV­(b) of IUPAC 2015 were considered, but these isotherms were indistinctly denoted as Type IV in their classification. Similarly, Donohue and Aranovich denoted both Types V­(a) and V­(b) as Type V. They showed the hysteresis cycles getting smaller with increasing temperature until the isotherms became reversible, even for *T* < *T*_c_. Donohue and Aranovich proposed a new classification^11^ in 1998, in which Type VI of IUPAC 2015 appears as a particular case of Types II and III for very low temperatures.

It is worth noting that liquid adsorption is not considered in any of the aforementioned classifications. That can be explained when the saturation of the pore occurs when the bulk is a gas, i.e., for Types I, IV­(a), IV­(b), V­(a), and V­(b), as the amount adsorbed does not vary significantly when the bulk is a liquid for these types. From an experimental and practical point of view, distinct types of equipment are used for gas and liquid adsorption (and porosimetry), which emphasizes that standard classifications of physisorption isotherms are focused on gas adsorption, instead of providing an overall picture of adsorption and intrusion for any phase. Liquid intrusion has a practical interest when the amount confined can vary significantly for *P* > *P* ^0^, which typically occurs for nonwetting liquids like mercury. The only types of isotherms for which the pore is not saturated for *P* < *P* ^0^ are Types II, III, and VI, which is consistent with the use of liquid intrusion for the characterization of macropores. The practical differences between gas and liquid adsorption are also emphasized by the multiplicity of the theoretical approaches used to describe the different types of isotherms (e.g., the methods of Langmuir, BET, BJH, etc.). These methods are mostly empirical and specific to a given type of isotherm, which increases the difficulty of studying the mechanisms of physisorption of gases, liquids, and supercritical fluids from a more fundamental perspective.

### 2.2 DFT Framework

Classical DFT^16,33^ is an approach based on statistical physics that can be applied to determine the microscopic structure of confined fluids. In our current work, the system considered is a Mie fluid characterized at the microscopic scale by the intermolecular potential

$$u^{Mie}\left(r\right)=\varepsilon \frac{\lambda_{r}}{\lambda_{r}-\lambda_{a}}{\left(\frac{\lambda_{r}}{\lambda_{a}}\right)}^{\lambda_{a}/(\lambda_{r}-\lambda_{a})}\left[{\left(\frac{\sigma }{r}\right)}^{\lambda_{r}}-{\left(\frac{\sigma }{r}\right)}^{\lambda_{a}}\right]$$ 1

where ε is the dispersion energy of the fluid–fluid interactions, σ is the particle diameter, and λ_r_ and λ_a_ are the repulsive and attractive exponents of the Mie potential, respectively. We consider the particular case of the Lennard-Jones potential by setting λ_r_ = 12 and λ_a_ = 6. Other values of exponents can be used to model real compounds with radial interactions (e.g., methane, nitrogen, argon, etc.) within the DFT approaches^16^ combined with the SAFT-VR Mie^17^ EoS.

The system is confined within a slit pore described by an external potential expressed as

2 $$V^{ext}\left(z\right)=V_{fw}\left(z\right)+V_{fw}\left(H-z\right)$$

where *V*_fw_(*z*) and *V*_fw_(*H* – *z*) represent the fluid–wall interactions of two walls placed at *z* = 0 and *H*, respectively.

The fluid–wall potential (for a single wall) is a 2D-integrated Mie potential expressed as

3 $$\begin{array}{ll} V_{fw}\left(z\right)= & \varepsilon_{fw}\frac{\left(\lambda_{fw}^{r}-2\right)\left(\lambda_{fw}^{a}-2\right)}{\lambda_{fw}^{r}-\lambda_{fw}^{a}}\\ & \times \left(\frac{1}{\lambda_{fw}^{r}-2}{\left(\frac{\sigma_{fw}}{z}\right)}^{\lambda_{fw}^{r}-2}-\frac{1}{\lambda_{fw}^{a}-2}{\left(\frac{\sigma_{fw}}{z}\right)}^{\lambda_{fw}^{a}-2}\right) \end{array}$$

where ε_fw_ is the minimum of the potential, referred to as the intensity of the fluid–wall interaction, σ_fw_ = σ/2 is the distance between the center of a fluid particle of diameter σ and the wall (assuming that the wall does not have width), and λ_fw_^r^ = 12 and λ_fw_^a^ = 6 are the exponents of the fluid–wall interaction in the case of a Lennard-Jones interaction.

The confined fluid is an open system in contact with a bulk fluid, characterized by the temperature *T* and the chemical potential μ. The free energy *F* of the bulk fluid is given by the SAFT-VR Mie EoS, and the chemical potential is obtained from the standard thermodynamic relation $\mu ={\left(\frac{\partial F}{\partial N}\right)}_{T,V_{bulk}}$ , where *V*_bulk_ is a large volume of the bulk fluid. The fluid confined within the slit pore is described in the grand-canonical ensemble at temperature *T*, chemical potential μ, and pore volume *V* = ∫_pore_d ***r***. The density of the confined fluid is not constant because of the presence of the fluid–wall interactions, such that the grand potential Ω that characterizes the system in the grand-canonical ensemble is a functional of the density profile ρ­(***r***). In the classical DFT framework, we define the functional^16^

4 $$\Omega \left[\rho \right]=F\left[\rho \right]+\int \rho \left(r\right)\left(V^{ext}\left(r\right)-\mu \right)d^{3}r$$

where F is the intrinsic free-energy functional that describes the contributions of the fluid–fluid interactions of the confined fluid. This intrinsic free-energy functional is given by the SAFT-VR Mie DFT approximation^29^ that we have developed recently. This choice ensures that F corresponds to *F* everywhere when the external potential is set to zero everywhere, which ensures the thermodynamic consistency of the approach.

The equilibrium state of the confined fluid is obtained by minimizing the functional Ω­[ρ] as

5 $$\frac{\delta \Omega \left[\rho \right]}{\delta \rho \left(r\right)}=\frac{\delta F\left[\rho \right]}{\delta \rho \left(r\right)}+V^{ext}\left(r\right)-\mu =0$$

by using the functional derivatives of the classical DFT framework,^33^ such that the minimum of Ω­[ρ] equals the grand potential Ω at equilibrium. The intrinsic free energy functional is split into an ideal part (i.e., the contribution to the free energy when the fluid–fluid interactions are neglected) and an excess part (that takes into account the fluid–fluid interactions) as $F=F^{id}+F^{ex}$ . The ideal part is given by $\beta F^{id}=\int \rho \left(r\right)\left[\ln\left(\rho \left(r\right)\Lambda^{3}\right)-1\right]d^{3}r$ , where $\beta =\frac{1}{k_{B}T}$ (with the Boltzmann constant *k*_B_) and Λ is the de Broglie thermal wavelength. The functional derivative $\frac{\delta \beta F^{id}}{\delta \rho \left(r\right)}=\ln\left(\rho \left(r\right)\Lambda^{3}\right)$ is used to obtain

6 $$\ln\left(\rho \left(r\right)\Lambda^{3}\right)+\frac{\delta \beta F^{ex}}{\delta \rho \left(r\right)}+\beta V^{ext}\left(r\right)-\beta \mu =0$$

The density profile at equilibrium can then be expressed as the self-consistent equation

7 $$\rho \left(r\right)=\frac{e^{\beta \mu }}{\Lambda^{3}}\exp\left(-\frac{\delta \beta F^{ex}}{\delta \rho \left(r\right)}-\beta V^{ext}\left(r\right)\right)$$

which is an explicit function of the chemical potential μ. It is convenient to consider the total amount *N*, i.e., the average number of particles found in the pore (which can be a noninteger number for an open system), as

8 $$N=\int_{pore}\rho \left(r\right)d^{3}r=\frac{e^{\beta \mu }}{\Lambda^{3}}\int_{pore}\;\exp\left(-\frac{\delta \beta F^{ex}}{\delta \rho \left(r\right)}-\beta V^{ext}\left(r\right)\right)d^{3}r$$

The self-consistent equation that gives the density profile is finally expressed as

9 $$\rho \left(r\right)=N\frac{\exp\left(-\frac{\delta \beta F^{ex}}{\delta \rho \left(r\right)}-\beta V^{ext}\left(r\right)\right)}{\int_{pore}\;\exp\left(-\frac{\delta \beta F^{ex}}{\delta \rho \left(r\right)}-\beta V^{ext}\left(r\right)\right)d^{3}r}$$

as an explicit function of the total amount *N* instead of the chemical potential μ. We solve eq 9 by iteration to find the density profile ρ­(***r***) at a given temperature *T*, pore volume *V*, and total amount *N*. In our current work, we prefer using the formulation in terms of the average number of particles in the pore *N* (eq 9) instead of μ (eq 7) because it is easier to study the hysteresis cycles of the adsorption isotherms: for a cycle, several values of *N* can be found for a given μ (which can cause numerical difficulties^34^), while only one value of μ can be found for a given value of *N* (counterexamples can be found in the literature when the internal states of the hysteresis cycle are studied, e.g., because of local multilayer adsorption,^35,36^ which is out of the scope of our current study). By solving eq 9, we obtain the stable and metastable states of the hysteresis cycle. We distinguish these states by comparing their grand potentials as the stable state is found for the minimum of the grand potential. For a given *N*, the chemical potential is known from eq 8

10 $$\beta \mu =\ln\left[\left(N\Lambda^{3}\right)/\int_{pore}\;\exp\left(-\frac{\delta \beta F^{ex}}{\delta \rho \left(r\right)}-\beta V^{ext}\left(r\right)\right)d^{3}r\right]$$

When the density profile at the microscopic scale is determined, other thermodynamic properties can be obtained, including those at the macroscopic scale. For the bulk fluid in contact with the system, the bulk density ρ and the bulk pressure *P* can be found from the chemical potential μ using standard thermodynamic relations and the free energy of SAFT-VR Mie. An equilibrium can be found between two phases of the bulk fluid when the pressures of the two phases are equal and when the chemical potentials of the two phases are also equal. Such an equilibrium corresponds to a VLE as the bulk fluid is pure, and solid–liquid equilibria in the bulk are not considered in our work.

The adsorbed amount (excess adsorption), $\Gamma =\frac{N-\rho V}{A}$ , can be used to represent adsorption isotherms, where *A* is the Gibbs dividing surface (which can be defined as the surface area of a single wall or as the sum of the surface areas of the two walls of the slit pore). The adsorbed amount is thus proportional to the difference between the average number of fluid particles confined in the pore, *N*, and the number of particles in a volume *V* of the bulk fluid with a number density ρ, *N*_bulk_ = ρ*V*. Similarly, the surface tension of the system can be expressed as $\gamma =\frac{\Omega +PV}{A}$ , which is proportional to the difference between the grand potential Ω of the confined fluid (obtained with eq 4) and the grand potential of the bulk fluid in the volume *V*, Ω_bulk_ = −*PV*.

It is convenient to use scaled properties to compare the adsorption isotherms of systems confined within pores of different sizes. That includes the bulk packing fraction $\eta =\frac{\pi }{6}\rho \sigma^{3}$ , the packing fraction of the confined fluid $\eta^{conf}=\frac{\pi }{6}\frac{N}{V}\sigma^{3}$ , and the excess packing fraction η^ex^ = η^conf^ – η (which is proportional to Γ). Other dimensionless properties used in our work are defined in Table 1, using diameter σ and dispersion energy ε as scales.

**Table 1:** List of Properties Considered, Together with the Scaling Convention Used in Our Current Work

property	standard property	dimensionless property
temperature	*T*	T* = *k*_B_ *T*/ε
pore width	*H*	H* = *H*/σ
fluid–wall interaction energy	ε_fw_	ε* = ε_fw_/ε
bulk density	ρ	$\eta =\frac{\pi }{6}\rho \sigma^{3}$ (bulk packing fraction)
bulk pressure	*P*	P* = βPσ^3^
chemical potential	μ	μ* = βμ (using Λ = σ)
total amount in the pore	*N*	$\eta^{conf}=\frac{\pi }{6}\frac{N}{V}\sigma^{3}$ of the confined fluid (packing fraction)
adsorbed amount	$\Gamma =\frac{N-\rho V}{A}$	η^ex^ = η^conf^ – η (excess packing fraction)
grand potential	Ω	$\Omega^{*}=\frac{\beta \Omega \sigma^{3}}{V}$
surface tension	$\gamma =\frac{\Omega +PV}{A}$	γ* = Ω* + P*

### 2.3 Topologies of Isotherms

We use classical DFT to revisit the influence of the temperature T*, pore size H*, and intensity of the fluid–wall interactions ε* on the shape of the isotherms of physisorption by considering the confinement of gases, liquids, and supercritical fluids over a wide range of thermodynamic conditions. In that sense, our study for a pure fluid in confinement can be seen as inspired by the classification of IUPAC 2015 ^31^ and by the work of Balbuena and Gubbins^10^ (analogously, we also mention the work of van Konynenburg and Scott for binary van der Waals mixtures^37^). For subcritical temperatures, Balbuena and Gubbins found Types I, III (referred to as III or VII in their work), IV­(a), IV­(b) (referred to as II), V­(a) (referred to as V), and VI; however, the standard Type II of IUPAC 2015, expected at quite low temperature for macropores, was missing from their study.^10^ We assume that this absence can be explained by the fact that Balbuena and Gubbins focused on the study of confined fluids at equilibrium instead of considering both stable and metastable states to find the full hysteresis cycles of the isotherms. The effects of temperature must also be detailed to understand the conditions of observation of Types IV­(b) and V­(b). This aspect is directly related to the possible topologies of the isotherms; e.g., we need to explain how an isotherm that contains a hysteresis cycle is modified by an increase of temperature to obtain an isotherm for a supercritical fluid that does not contain a hysteresis cycle.

The topologies considered in our work are listed in Figure 2. The curves can represent any property defined for the confined fluid (e.g., the total packing fraction, excess packing fraction, grand potential, and surface tension) as a function of any property considered for the bulk fluid (e.g., the bulk packing fraction, pressure, or chemical potential). All the curves have two end points e and s that characterize the empty pore and the saturated pore, respectively.

**Figure 2:**
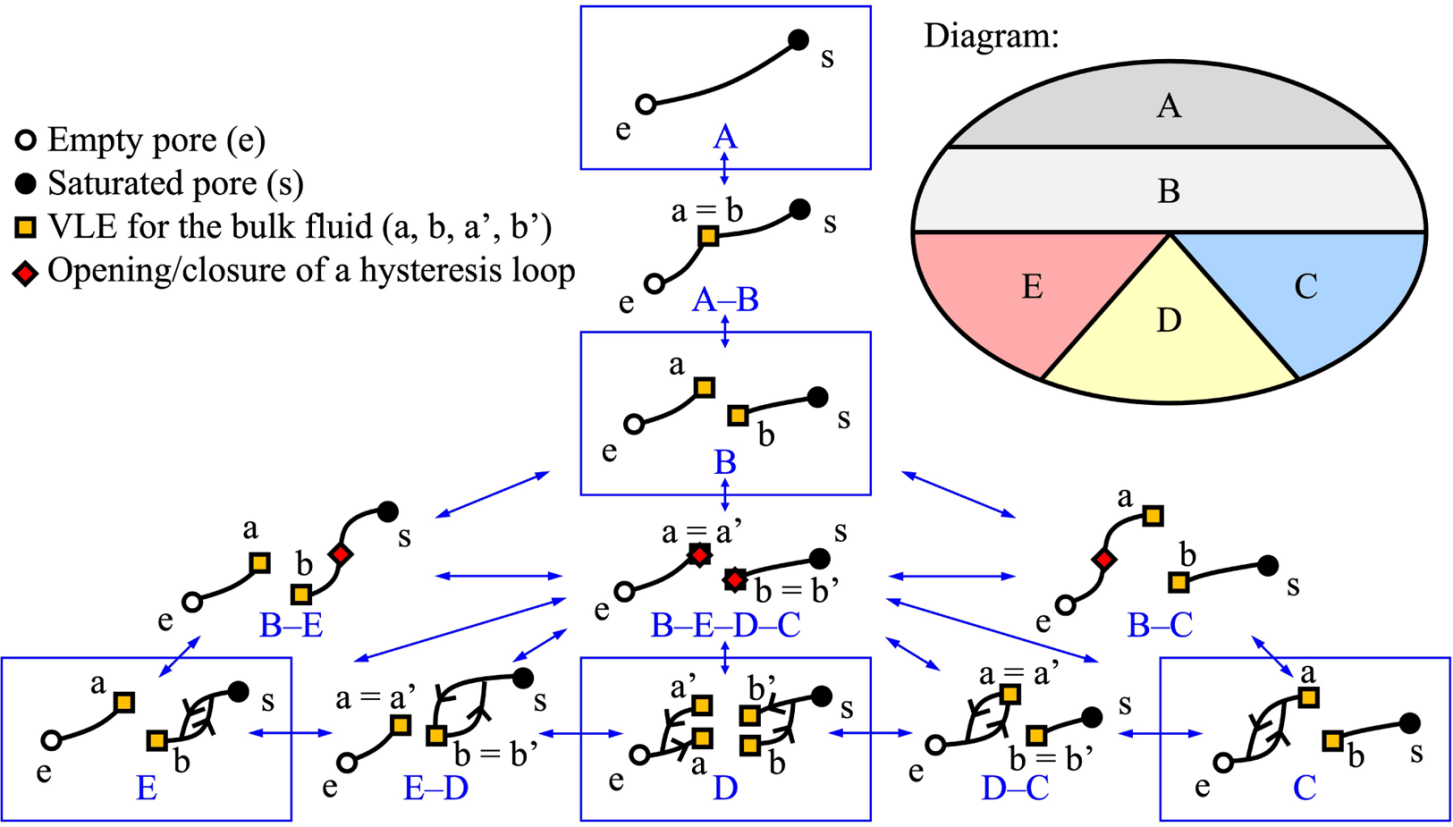
Possible topologies of physisorption isotherms, considering the vapor–liquid equilibrium (VLE) of the bulk fluid and the capillary condensation/evaporation of the confined fluid (as a unique hysteresis cycle).

The simplest topology, denoted as **Topology A**, consists of a curve between points e and s, which represents both adsorption and desorption curves. That means that the isotherm is reversible, and each property varies continuously. This topology corresponds to supercritical fluids as there is no phase separation for both the bulk fluid and the confined fluid. When the temperature corresponds to *T*_c_, the critical point related to the bulk fluid must appear on the curve (cf. Topology A–B in Figure 2).

For *T* < *T*_c_, the bulk packing fraction is not continuous. This is represented by a split of the curve into two parts, as can be seen for **Topology B**: from point e to point a, the bulk fluid is a gas; from point b to point s, the bulk fluid is a liquid. At equilibrium, the bulk pressure and the chemical potential are equal to *P* ^0^ and μ^0^, respectively, for both points a and b of Topology B. This means that the curve split indicates that there is a discontinuity for at least one property, but it does not mean that all the properties are discontinuous. This also means that there is a VLE for the bulk fluid for Topology B, but there is no phase separation for the confined fluid, which behaves like a supercritical fluid (i.e., there is no hysteresis cycle).

By contrast, we consider that there is a phase separation for the confined fluid for Topologies C, D, and E (which always occurs when there is a VLE for the bulk fluid due to the fluid–wall interactions). As we consider both stable and metastable states for the phase separation of the confined fluid, we represent the opening/closure of a hysteresis cycle on a part of a curve of Topology B (cf. Topologies B–C, B–E–D–C, and B–E in Figure 2) to explain the transition between Topology B and Topologies C, D, and E. The irreversibility of an isotherm implies that the mechanisms of adsorption and desorption are different, which is indicated by the arrows on the hysteresis cycles. **Topology C** is obtained when the hysteresis cycle is found between points e and a (which can be obtained from Topology B through Topology B–C). This means that the confined fluid can exist as a gas or a condensed phase, while the bulk remains a gas, which corresponds to capillary condensation. By analogy, **Topology E** is obtained when the hysteresis cycle is found between points b and s (e.g., from Topologies B and B–E). This case corresponds to capillary evaporation and liquid intrusion.

In **Topology D**, the hysteresis cycle exists on both sides of the points that represent the VLE of the bulk, i.e., points a, b, a’, and b’. The part of the isotherm that corresponds to adsorption is composed of points e, a, b, and s, which indicates that the condensation of the confined fluid occurs when the bulk is a liquid (such as topology E). The part of the isotherm that corresponds to desorption is composed of points s, b’, a’, and e, which indicates that the evaporation of the confined fluid occurs when the bulk is a gas (like topology C). In other words, for Topology D, both capillary condensation and evaporation are delayed compared to the VLE of the bulk. Topology D can be found from Topologies C and E by increasing the size of the hysteresis cycle until reaching points a and b. Alternatively, Topologies B, B–C, C, D–C, D, E–D, E, and B–E can be found from Topology B–E–D–C, which represents the opening/closure of a hysteresis cycle on the exact points a and b.

It is apparent from Figure 2 that the five Topologies A to E can be represented as surfaces of an arbitrary two-dimensional diagram. Due to the number of degree of freedom (or variance) that characterizes each topology, the five Topologies A–B, B–C, B–E, D–C, and D–E can be represented as curves on the diagram, and Topology B–E–D–C can be represented as a point. On the diagram, Topology A has a mutual border with Topology B but not with Topologies C, D, and E. Topologies B and D cannot be separated by a curve but can be separated only by the point of Topology B–E–D–C. Similarly, Topologies C and E are only joined by the point of Topology B–E–D–C.

When the possible phase transitions considered are limited to the VLE of the bulk fluid and the presence of a unique hysteresis cycle, the topological features represented in Figure 2 must be satisfied for any phase diagram characterized by two axes, e.g., using T*–ε*, H*–ε*, or T*–H* diagrams. This feature is used to define the boundaries of our study. Temperatures T* must be considered from low values (above the freezing point), for which one and only one hysteresis cycle can be found (cf. Topologies C, D, and E), to high values, where the fluid is supercritical (cf. Topology A). We find that this range can be 0.5 ≤ T* ≤ 1.4 for the Lennard-Jones systems considered. The range of pore sizes H* must cover micropores, mesopores, and macropores. However, for macropores, we do not detail phenomena that go beyond the scope defined by the topological features described in Figure 2 (e.g., the wetting transition^33^ and local multilayer adsorption^35,36^ ), and we find that we only need to consider 1 ≤ H* ≤ 15. The intensity of the fluid–wall interaction ε* must be large enough to find all types of physisorption isotherms, and we consider 0 ≤ ε* ≤ 16.

## 3 Results and Discussion

### 3.1 Hysteresis Cycles for Large Mesopores and Macropores

We provide examples of physisorption isotherms that can be used to explain transitions from a given type of isotherm to another type by changing temperature T*, pore size H*, or the fluid–wall interaction energy ε*. We start by considering an example of Type IV­(a), which can be typically found for mesopores with strong fluid–wall interactions as the first layers of adsorption are formed at low pressure. For the system under investigation, we find such an isotherm for T* = 0.85, ε* = 8.0, and H* = 10.0. The two branches of the hysteresis cycle are found for *P* < *P* ^0^, as can be seen in Figure 3a, when the packing fraction of the confined fluid η^conf^ is represented as a function of P*. We then keep T* and ε* constant, and we consider a slightly wider pore, for H* = 11.0. For *P* < *P* ^0^, the adsorption curve does not reach saturation and exhibits the characteristic sigmoid shape of Type II, but this only represents a small part of the full isotherm. For *P* > *P* ^0^, this adsorption curve continues increasing with an increase of the bulk pressure, and a phase transition that corresponds to the condensation of the confined fluid is observed. We also note from Figure 3a that the desorption curves for H* = 10.0 and 11.0 are similar. That means that the full isotherm observed for H* = 11.0 has a Topology D, and we denote this case as **Type II–IV**. To summarize, we observe a transition from Topology C (with Type IV­(a)) to Topology D (with Type II–IV) by increasing the pore size. The full isotherm of Type II–IV has a large hysteresis cycle, for which the adsorption and desorption branches are spread on both sides of *P* ^0^. If only gas adsorption is considered, for *P* < *P* ^0^, the condensation of the confined fluid may not be reached (no hysteresis cycle would be visible), and the isotherm would look like the standard Type II instead of Type II–IV. Similar conclusions can be established when η^conf^ is represented as a function of the chemical potential μ*, as can be seen in Figure 3b.

**Figure 3:**
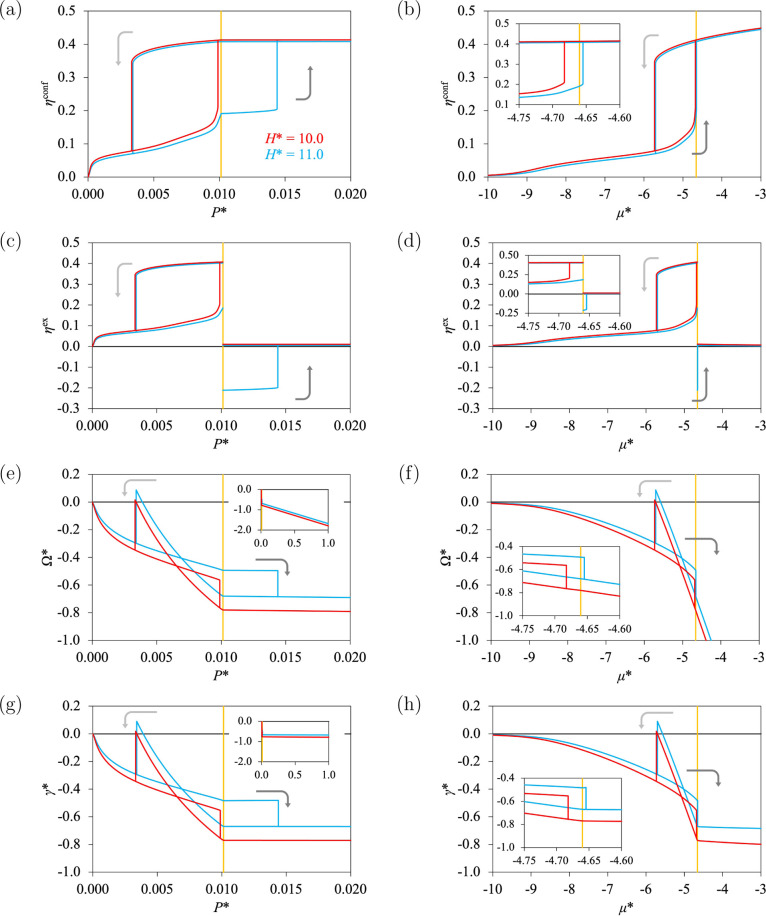
Adsorption isotherms at T* = 0.85 and ε* = 8.0 as functions of pressure P* (left) and chemical potential μ* (right) for H* = 10.0 (red) and 11.0 (blue). The vapor-liquid equilibrium of the bulk fluid is represented by vertical gold curves. The arrows indicate the adsorption (light gray) and desorption (dark gray) branches. (a,b) Packing fraction of the confined fluid. (c,d) Excess packing fraction. (e,f) Grand potential. (g,h) Surface tension.

We also show the excess packing fraction η^ex^ (proportional to the adsorbed amount) in Figure 3c,d as it is the standard quantity considered in adsorption studies for gas adsorption. It is clear that η^ex^ is discontinuous at *P* ^0^ when both gas adsorption and liquid intrusion are considered (because of the discontinuity of the bulk packing fraction η).

We now determine the conditions of equilibrium of the phase transition found for the confined fluid. To do so, we consider the grand potential Ω*, shown in Figure 3e,f as a function of P* and μ, respectively. For the hysteresis cycles, two values of Ω* are found at a given value of μ*: one corresponds to the stable state of the system (for the minimum of Ω*) and the other one corresponds to a metastable state. The intersection between the adsorption and desorption curves, as shown in Figure 3e,f, denotes the condition of equilibrium related to the phase transition under confinement. This equilibrium (eq) is found for *P*_eq_ < *P* ^0^ and μ_eq_ < μ^0^, for both H* = 10.0 and 11.0, which means that Type II–IV looks like Type IV­(a) if only the equilibrium is considered, instead of considering the hysteresis cycle (the absence of Type II at low temperature in the work of Balbuena and Gubbins^10^ is explained by this behavior). It also means that the difference between Types IV­(a) and II–IV (i.e., the standard Type II for gas adsorption) is due to the size of the hysteresis cycle, driven by dynamical effects, and is not due to the conditions of thermodynamic equilibrium. We choose to refer to this behavior as Type II–IV to emphasize the dynamical effects, which is consistent with the experimental practice and the observation of Type II for gas adsorption (an alternative name like Type “IV­(II)” would emphasize the behavior at equilibrium).

Similar conclusions can be found from the surface tension calculations presented in Figure 3g,h. We also recall that Topologies C and D, as defined in Section 2.3, can be seen for any subfigure of Figure 3.

By analogy with the features described for new Type II–IV, shown in Figure 3, we define a new Type III–V, which can be found for weaker fluid–wall interactions (i.e., when there is no formation of the first layers of adsorption at low pressure). Both Types II–IV and III–V are represented in Figure 4 for comparison. Type III–V can be found by considering larger pore sizes from the standard Type V­(a), which constitutes another example of transition from Topology C to Topology D. The full hysteresis cycle cannot be seen when the study is limited to gas adsorption, i.e., for *P* < *P* ^0^, and Type III–V should appear like Type III of IUPAC 2015 in that case.

**Figure 4:**
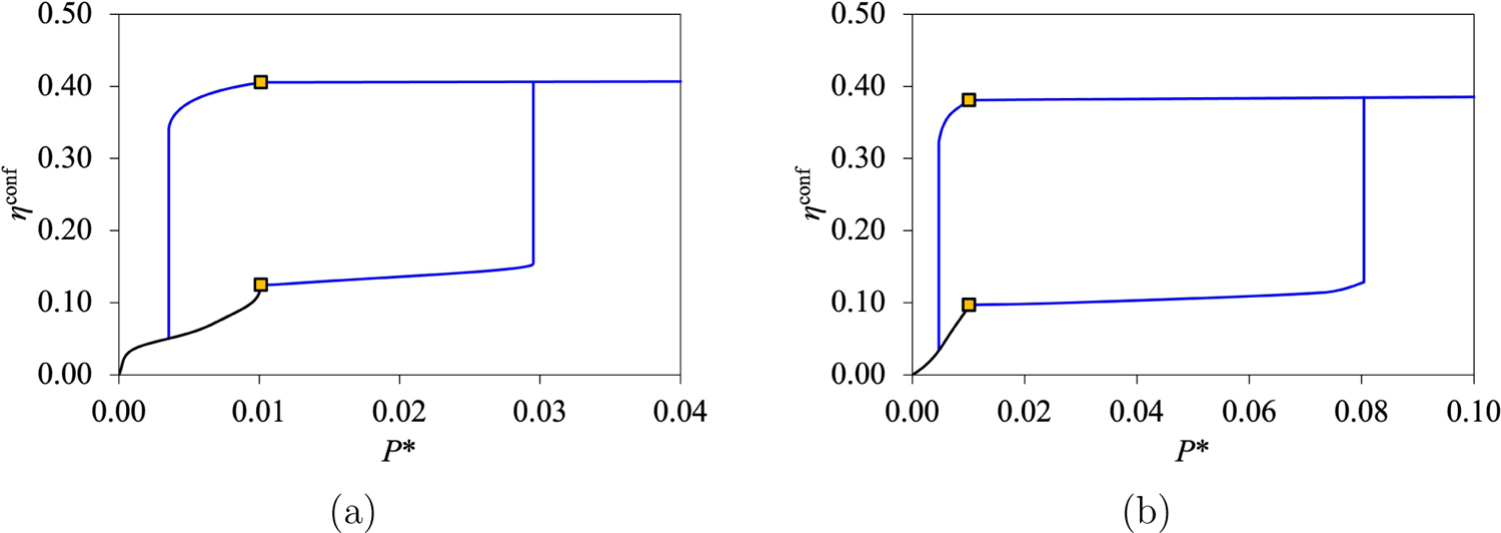
Packing fraction of the confined fluid η^conf^ as a function of pressure P*. The black curves represent the part of the isotherms obtained with gas adsorption. The blue curves represent the other parts of the full isotherms, obtained by considering the vapor–liquid equilibrium of the bulk fluid in contact with the confined system. The gold squares indicate the saturation of the bulk fluid (at vapor pressure *P* ^0^) for the two branches of the isotherm. (a) Type II–IV, obtained for strong fluid–wall interactions with T* = 0.85, H* = 15.0, and ε* = 7.5. (b) Type III–V, obtained for weak fluid–wall interactions with T* = 0.85, H* = 5.0, and ε* = 4.5.

For the systems considered in our work, Type III–V can be found for T* = 0.85, H* = 5.0, and ε* = 4.5, as shown in Figure 4b. For these thermodynamic conditions, the equilibrium of the phase transition for the confined fluid, characterized by *P*_eq_, can be found for *P*_eq_ < *P* ^0^ (like Type V­(a)). By contrast, for even lower fluid–wall interactions, this equilibrium can be found for *P*_eq_ > *P* ^0^, e.g., for T* = 0.85, H* = 5.0, and ε* = 3.0. These two cases can be analyzed with classical DFT by considering the grand potential, and the new types of adsorption isotherm could correspondingly be referred to by different names, e.g., Types “V­(III)” and “III­(V)” for *P*_eq_ < *P* ^0^ and *P*_eq_ > *P* ^0^, respectively. However, Types “V­(III)” and “III­(V)” would not be distinguishable experimentally, and we prefer to denote both of them as Type III–V in our work for that reason.

One could expect Type II–IV to be split into two similar cases, which would have been referred to as Type “IV­(II)” for *P*_eq_ < *P* ^0^ and Type “II­(IV)” for *P*_eq_ > *P* ^0^ from a theoretical point of view. However, for the systems considered in our work, the formation of the first layer at low pressure is systematically found for *P*_eq_ < *P* ^0^, which corresponds to Type “II­(IV)” only. The hypothetical Type “II­(IV)” may exist for other systems (i.e., different models of fluid and wall), but searching for these types of isotherms is out of the scope of our current work.

### 3.2 Isotherms for Micropores

We consider several pore sizes, from H* = 1.5 to 4.0, to investigate the transition from isotherms of Type I (with Topology B), typically found for micropores, to isotherms of Type IV­(a) (with Topology C), typically found for mesopores. The temperature and fluid–wall interaction energy are set to T* = 0.7 and ε* = 12.0, respectively, and we show the isotherms in Figure 5 as functions of pressure P* or chemical potential μ*.

**Figure 5:**
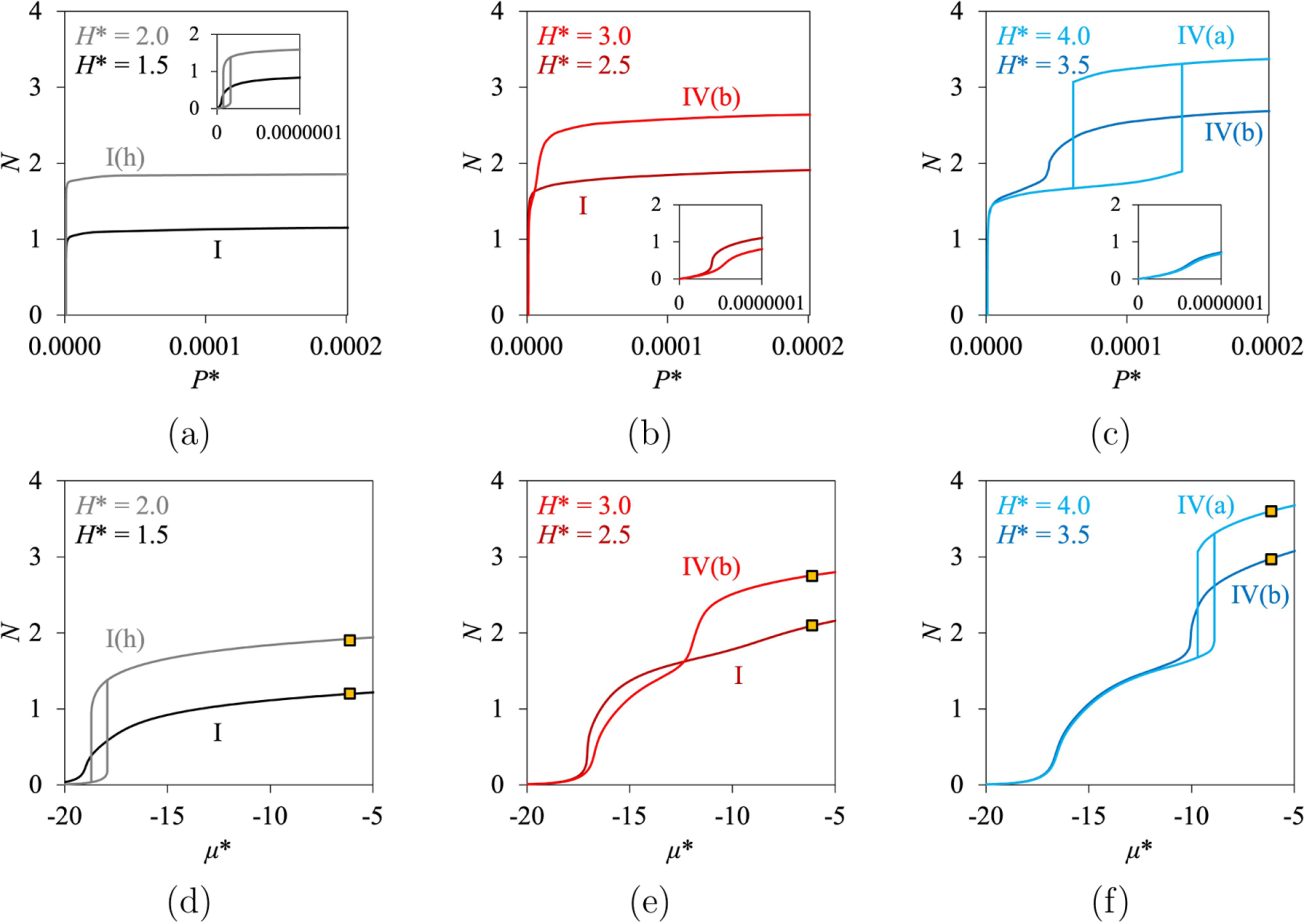
Average number of molecules *N* (proportional to η^conf^) as a function of pressure P* (top) and chemical potential μ* (bottom), at T* = 0.7 and ε* = 12.0, for (a,d) narrow micropores, (b,e) larger micropores, and (c,f) small mesopores. The gold squares indicate the phase transition of the bulk fluid.

As expected, Type I is found for the smallest pore size considered, i.e., H* = 1.5 (Figure 5a,d), with a sharp increase at very low pressure. Such an isotherm is referred to as Type I­(a) in IUPAC 2015 or simply as Type I in other classifications and in our current work. A smoother Type I is found for H* = 2.5 (Figure 5b,e), referred to as Type I­(b) in IUPAC 2015 (and as Type I elsewhere). For H* = 3.0 and 3.5 (shown in Figure 5b, c, e, f), several layers of adsorption can fit into the pore (i.e., the saturation is not reached after the formation of the first layer, in contrast with Type I), and the isotherms obtained correspond to Type IV­(b) as they are reversible. Such a result is consistent with the indications of IUPAC 2015, which mentions that Type IV­(b) can be found for small mesopores. Type IV­(a) is found for the largest pore size studied in this section, for H* = 4.0 (Figure 5c,f), as the isotherm is not reversible. It appears that for strong fluid–wall interactions, the first layer of adsorption is always formed at low pressure, independent of the pore size, while capillary condensation only occurs when the pore is large enough, for higher pressures, after the formation of the first layer. From a topological point of view, this means that Type IV­(b) must be found systematically between Types I and IV­(a), by considering different pore sizes. Such a statement should be true for any pore geometry (even if it might be easier to find Type IV­(b) for conical and cylindrical geometries rather than for slit pores, as suggested by IUPAC 2015 ^1^).

Uncommon features are found for H* = 2.0. The isotherm looks like a sharp Type I when the amount confined in the pore is represented as a function of pressure (Figure 5a), i.e., the pore is saturated by the formation of the first layer at very low pressure. However, the formation of the first layer is not predicted as reversible, in contrast with the standard description of Type I. A tiny hysteresis cycle is obtained with the classical DFT calculations, which can be seen in the inset of Figure 5a, and for very low values of chemical potential, shown in Figure 5d. We denote this potentially new type of isotherm as **Type I­(h)**. By considering extremely low values of pressure, Types I­(h) and I are qualitatively and topologically similar to Types V­(a) and V­(b), respectively. However, the respective inflection points of Types I and V­(b) can be distinguished quantitatively when the isotherms are expressed as a function of pressure, as can be seen in Figure 5a. In practice, the pressure of the inflection point of Type I is so small that the isotherms look concave. Similarly, the hysteresis cycle of Type I­(h) is commonly neglected because of its size on the pressure scale, contrary to the hysteresis cycle of Type V­(a). When the isotherms are expressed as a function of the chemical potential, as in Figure 5d, the hysteresis cycle of Type I­(h) does not look negligible due to the effects of scale related to pressure and chemical potential.

If isotherms of Type I­(h) exist for real materials, they would be difficult to observe due to the range of pressure that would have to be investigated to determine the adsorption and desorption branches of the hysteresis cycle, which may be inaccessible because of the limitations of the equipment or hidden by the experimental uncertainty. It is also possible that the hysteresis cycle of Type I­(h) may be affected by the roughness of the pore surface, which may make the study of Type I­(h) for real materials even more complex.

### 3.3 Effect of Temperature

In Figure 6, we represent the effect of the temperature on the shape of physisorption isotherms for several combinations of pore size H* and fluid–wall interaction energy ε*.

**Figure 6:**
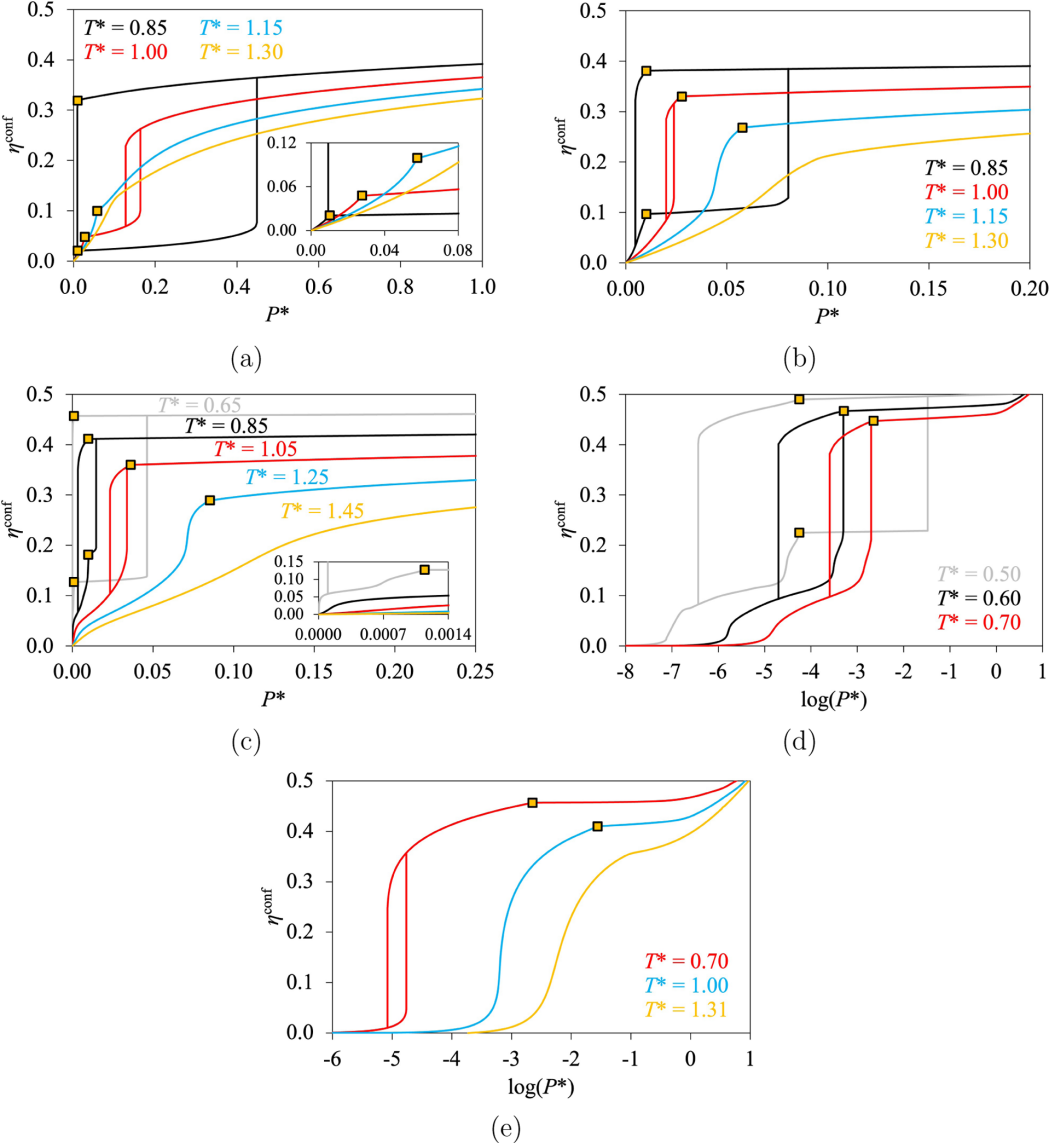
Physisorption isotherms for various thermodynamic conditions. The gold squares indicate the phase transition of the bulk fluid. (a) Types III–V in black, III­(a) in red, III­(b) in blue, and III(s) in gold for H* = 4.0 and ε* = 3.0. (b) Types III–V in black, V­(a) in red, V­(b) in blue, and III(s) in gold for H* = 5.0 and ε* = 4.5. (c) Types VI–IV in gray, II–IV in black, IV­(a) in red, IV­(b) in blue, and II(s) in gold for H* = 12.0 and ε* = 8.0. (d) Types VI–IV in gray, VI­(a) in black, and IV­(a) in red for H* = 7.0 and ε* = 8.0. (e) Types I­(h) in red, I in blue, and I(s) in gold for H* = 2.0 and ε* = 7.0.

In Figure 6a, we show isotherms for H* = 4.0 (mesopore) and ε* = 3.0 (corresponding to very weak fluid–wall interactions). Type III–V is found at low temperature (T* = 0.85) with a large hysteresis cycle. The equilibrium of the phase transition for the confined fluid is found at a pressure *P*_eq_ that is larger than *P* ^0^ (i.e., this Type III–V could be referred to as Type “III­(V)” from a theoretical point of view). The size of the hysteresis cycle decreases with an increase in temperature such that the irreversible part of the isotherm can be fully found for *P* > *P* ^0^, when the bulk is a liquid (e.g., for T* = 1.00). We refer to this phenomenon as capillary evaporation, and we denote this type of isotherm as Type III­(a). Such a type is typically found for liquid intrusion and techniques of mercury porosimetry.^3,38–40^ The hysteresis cycle can be closed by increasing the temperature (T* = 1.15). We refer to this type of reversible isotherm as Type III­(b). It is important to note that Types III–V, III­(a), and III­(b) correspond indistinctly to Type III of IUPAC 2015 for gas adsorption, if only the range *P* < *P* ^0^ is considered. The potential irreversibility of an isotherm of Type III is revealed only when liquid adsorption/intrusion is included in the study. For supercritical temperatures (for T* = 1.30 and above), the isotherm is continuous and we refer to it as Type III(s) as the characteristic convex shape of Type III is still found at low pressure. To summarize, Types III–V, III­(a), III­(b), and III(s), shown in Figure 6a and obtained by increasing temperature, have Topologies D, E, B, and A, respectively.

An alternative set of isotherms from Type III–V to Type III(s) is shown in Figure 6b, for H* = 5.0 (mesopore) and ε* = 4.5 (weak fluid–wall interactions). Type III–V is found at very low temperature, but the equilibrium of the phase transition is found for *P*_eq_ < *P* ^0^ (i.e., this Type III–V could be denoted as Type “V­(III)” in theory). By increasing the temperature from this Type III–V, we find Types V­(a), V­(b), and III(s) (for T* = 0.85, 1.00, 1.15, and 1.30, respectively), which correspond to Topologies D, C, B, and A, respectively.

For H* = 12.0 (macropore) and ε* = 8.0 (strong fluid–wall interactions), the first layers of adsorption are systematically found at low pressure, as can be seen in Figure 6c. At very low temperature (T* = 0.65), we also observe the formation of successive layers for *P* < *P* ^0^, which corresponds to Type VI of IUPAC 2015. However, in contrast to the common description of Type VI, a desorption curve (similar to the desorption curve of Type IV­(a)) is also found for this isotherm. This corresponds to a hysteresis cycle found on both sides of *P* ^0^, which corresponds to Topology D, and we denote this behavior as Type VI–IV. From this Type VI–IV isotherm, we successively find Types II–IV (T* = 0.85), IV­(a) (T* = 1.05), and IV­(b) (T* = 1.25) by increasing the temperature to the critical temperature *T*_c_ of the bulk fluid. For supercritical temperatures (e.g., T* = 1.45), we can still observe the shape of Type IV­(b), with Topology A instead of B. We refer to this as Type II(s), for consistency with the work of Balbuena and Gubbins.^10^ The topologies shown in Figure 6c are Topologies D (for both T* = 0.65 and 0.85), C, B, and A.

An alternative transition from Type VI–IV to Type II(s) can be found with an increase in temperature, as can be seen in Figure 6d for H* = 7.0 (large mesopore) and ε* = 8.0 (strong fluid–wall interactions). We find that some isotherms can present successive layers (like Type VI of IUPAC 2015) and a full hysteresis cycle for *P* < *P* ^0^ (e.g., for T* = 0.60). We denote this as Type VI­(a), by analogy with Types V­(a) and IV­(a), which also have Topology C. By increasing T*, the successive layers of Type VI­(a) become less noticeable, and the isotherms are similar to the standard Type IV­(a), for which only the first layer remains at low pressure. The presence of Type VI­(a) or Type II–IV between Types VI–IV and IV­(a) depends on T*, H*, and ε*, which all affect the size of the hysteresis cycle and the ability of forming successive layers of adsorption.

In Figure 6e, we consider H* = 2.0 (micropore) and ε* = 7.0 (strong fluid–wall interactions). Types I­(h) and I are found for subcritical temperatures (as shown for T* = 0.70 and 1.00, respectively). For supercritical temperatures (for T* ≥ 1.30), we denote the isotherms found as Type I(s) as the pore is saturated by the formation of the first layers of adsorption at a low pressure. We use a logarithmic scale to represent the pressure axis in Figure 6e in order to clearly distinguish Topologies C, B, and A and to highlight the presence of the hysteresis cycle of Type I­(h).

### 3.4 New Classification of Physisorption Isotherms for Gases and Liquids

We propose a new classification of physisorption isotherms based on the results presented in our current work obtained with classical DFT. In Figure 7, we show the total amount *N* in the pore (or, equivalently, the packing fraction of the confined fluid η^conf^) as a function of the bulk pressure *P*. The classification is organized to emphasize topologies and effects of temperature, pore size, and the intensity of the fluid–wall interactions. For example, the types are placed along vertical axes according to their dependence in temperature.

**Figure 7:**
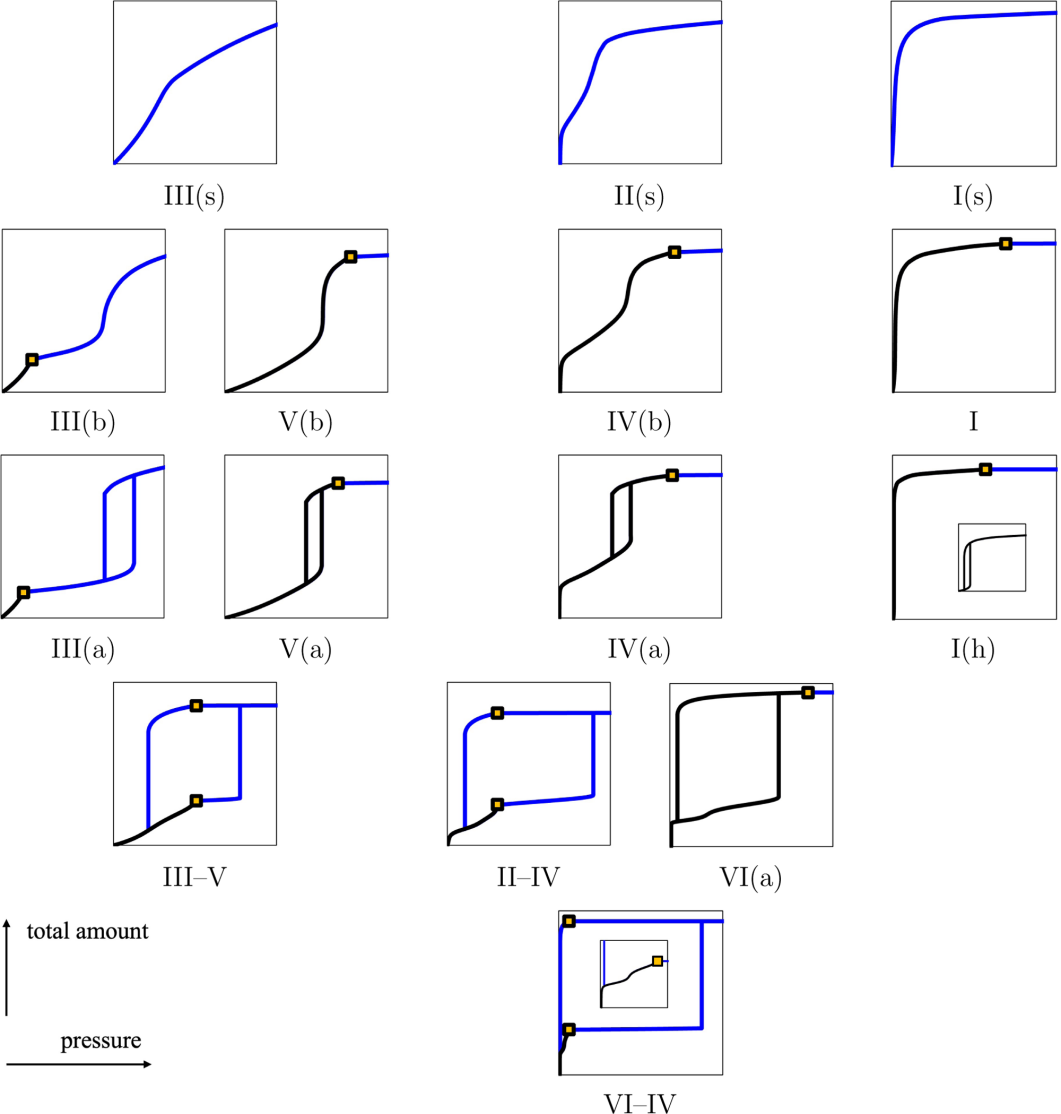
Proposed classification of physisorption isotherms for gases, liquids, and supercritical fluids, based on the results obtained in our current work with classical DFT. The total amount *N* in the pore (proportional to the packing fraction of the confined fluid η^conf^) is represented as a function of the bulk pressure *P*. The parts of the isotherms obtained by gas adsorption are shown in black. The other parts of the full isotherms are shown in blue. The gold squares indicate the saturation of the bulk fluid (at vapor pressure *P* ^0^).

An alternative representation of the classification, considering the amount adsorbed Γ (proportional to the excess packing fraction η^ex^) as a function of bulk pressure *P*, is shown in Figure 8. Considering the amount adsorbed Γ instead of the total amount *N* is common for gas adsorption, but such a choice introduces a discontinuity in Γ at the bulk vapor pressure *P* ^0^ for the subcritical isotherms.

**Figure 8:**
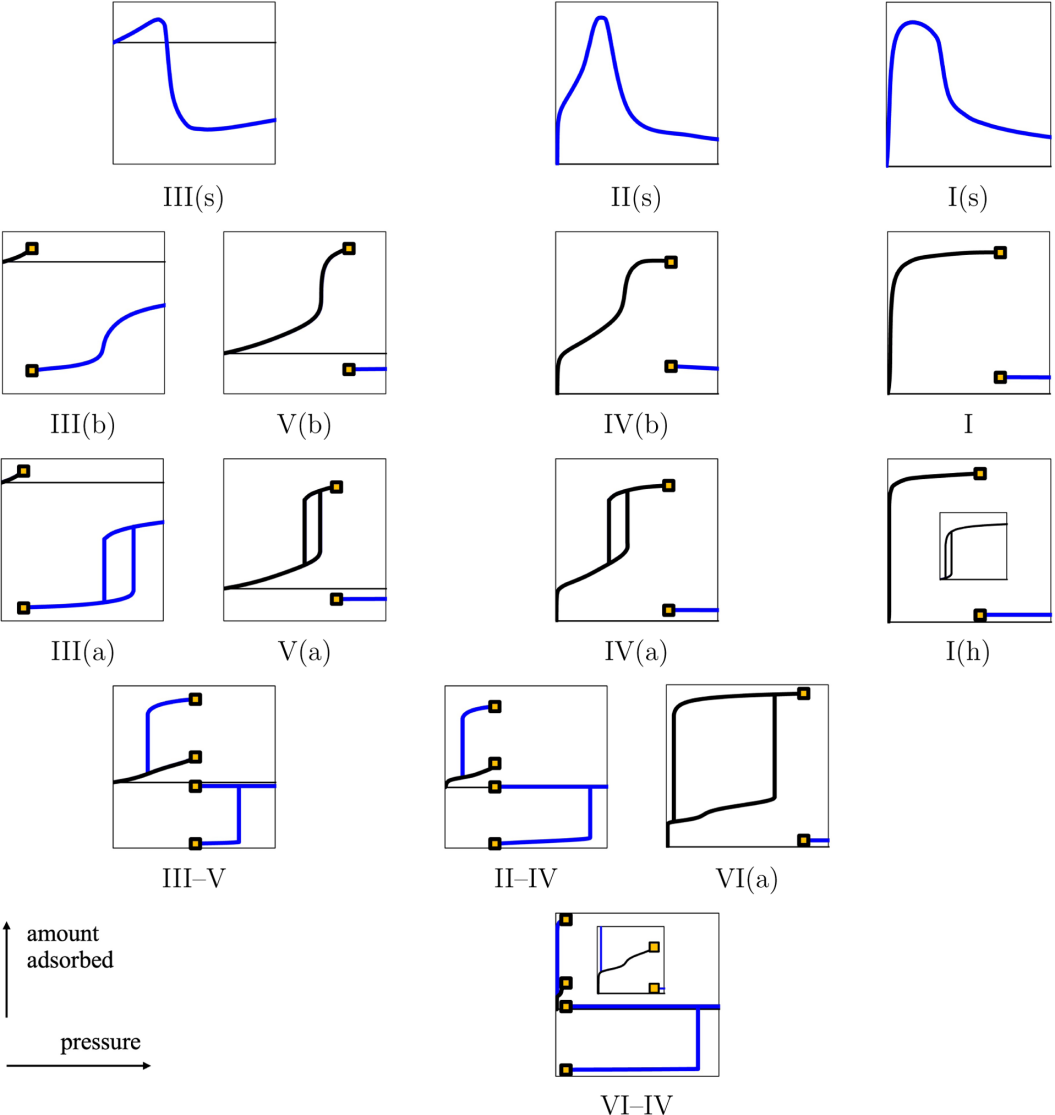
Same as Figure 7, now considering the amount adsorbed Γ (proportional to the excess packing fraction η^ex^) as a function of the bulk pressure *P*.

We show all of the types found in our study, but we do not imply that our classification is exhaustive for real systems as we consider only the confinement of a Lennard-Jones fluid between two planar walls. Other models of fluids and walls may introduce additional types, but these are outside the scope of our current work. For example, we can imagine an isotherm with a hysteresis cycle when the bulk is a liquid (like Type III­(a)), for which the first layers would be formed at low pressure (cf. Types II–IV, IV­(a), and IV­(b)); we denote such a hypothetical type as Type II­(a) and represent it in Figure 9. The hysteresis cycle of Type II­(a) would close with increasing temperature, and the isotherm obtained would be termed Type II­(b), as shown in Figure 9. We may also find combinations of multiple hysteresis cycles at very low temperature, as reported in previous studies,^10,41^ e.g., for substrates with complex geometries of pores.

**Figure 9:**
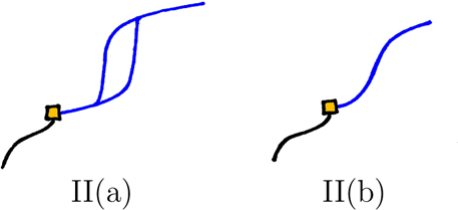
Sketches of the hypothetical Types II­(a) and II­(b). The total amount *N* (or the packing fraction of the confined fluid η^conf^) is represented as a function of the bulk pressure *P*. The curves, symbols, and colors have the same meaning as in Figure 7.

In Figure 10, we present phase diagrams at constant H* to show the topologies and types as functions of the temperature T* and fluid–wall interaction ε*. The diagrams are similar to the schematic representation of topologies shown in Figure 2. As expected, Topology A can be seen for supercritical temperatures (i.e., for *T* ≥ 1.3) and Topology B is systematically found for the highest subcritical temperatures. Topologies E, D, and C are found for the lowest temperatures, considering very low, low, and high values of ε*, respectively.

**Figure 10:**
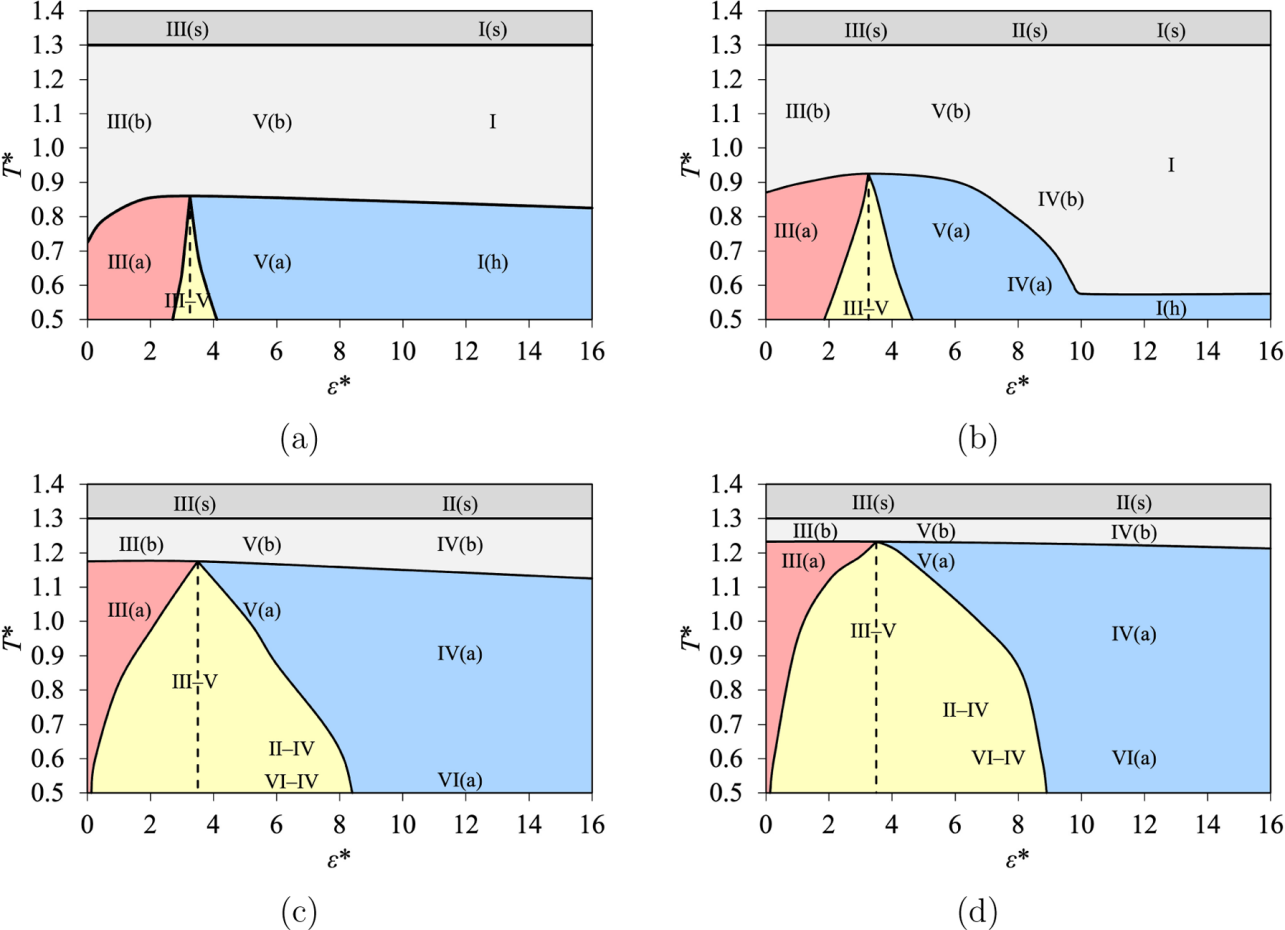
Phase diagrams at constant pore size H*, represented in terms of the temperature and the fluid–wall interaction energy (T*–ε* diagrams), for (a) H* = 2.25; (b) H* = 3.00; (c) H* = 7.50; and (d) H* = 12.0. The topologies of the isotherms are indicated with colored regions delimited by continuous curves for Topologies A (dark gray), B (light gray), C (blue), D (yellow), and E (red). The regions where capillary condensation and capillary evaporation can be found at equilibrium (for high and low ε*, respectively) are separated by dashed lines.

The size of the region occupied by Topology B is large for micropores (Figure 10a,b), smaller for mesopores (Figure 10c), and very small for macropores (Figure 10d). By contrast, the size of the region occupied by Topology D increases with an increase in H*, which reduces the size of the region occupied by Topology E. Topology C is always found for the largest values of ε*, such that the size of its region depends on the range of ε* shown in the diagram.

We also indicate the types defined in our classification in the phase diagrams shown in Figure 10. For low values of ε*, we systematically find Types III–V, III­(a), and V­(a) at low T* and Types III­(b), V­(b), and III(s) at higher T*. As shown in Figure 10a for H* = 2.25, Types I­(h), I, and I(s) are found for high values of ε*, and a continuous transition from Type I­(h) to Type V­(a) can be seen by reducing ε*. For the larger pore sizes, shown in Figure 10b,c,d for H* = 3.00, 7.50, and 12.0, respectively, Types IV­(a), IV­(b), and II(s) are also found for high to very high values of ε*. When the pore is large enough (i.e., for H* = 7.50 and 12.0), Types II–IV, VI–IV, and VI­(a) are also seen at low temperature.

An example of a H*–ε* diagram is provided in Figure 11 for T* = 0.7. Only Topologies B to E are found as the temperature is subcritical. The diagram is more complex than the schematic diagram shown in Figure 2 because Topology B occupies two nonconnected regions separated by a branch of Topology C (where Type I­(h) can be found). Such a feature is a consequence of the effects of confinement for small values of H* as the microscopic structure of the fluid and the formation of layers of adsorption are more sensitive to the space available in the pore. For Topology B, Types III­(b), V­(b), and I are found in the region characterized by the smallest values of H*, and Types V­(b), I, and IV­(b) are found in the region characterized by larger values of H* and high values of ε*. Types I­(h), V­(a), IV­(a), and VI­(a) are found in the region occupied by Topology C, while Type III­(a) is found in the region occupied by Topology E. As expected for Topology D, we find Types III–V (for low values of ε*), II–IV (for medium values of ε*), and VI–IV (for higher values of ε* and larger H*).

**Figure 11:**
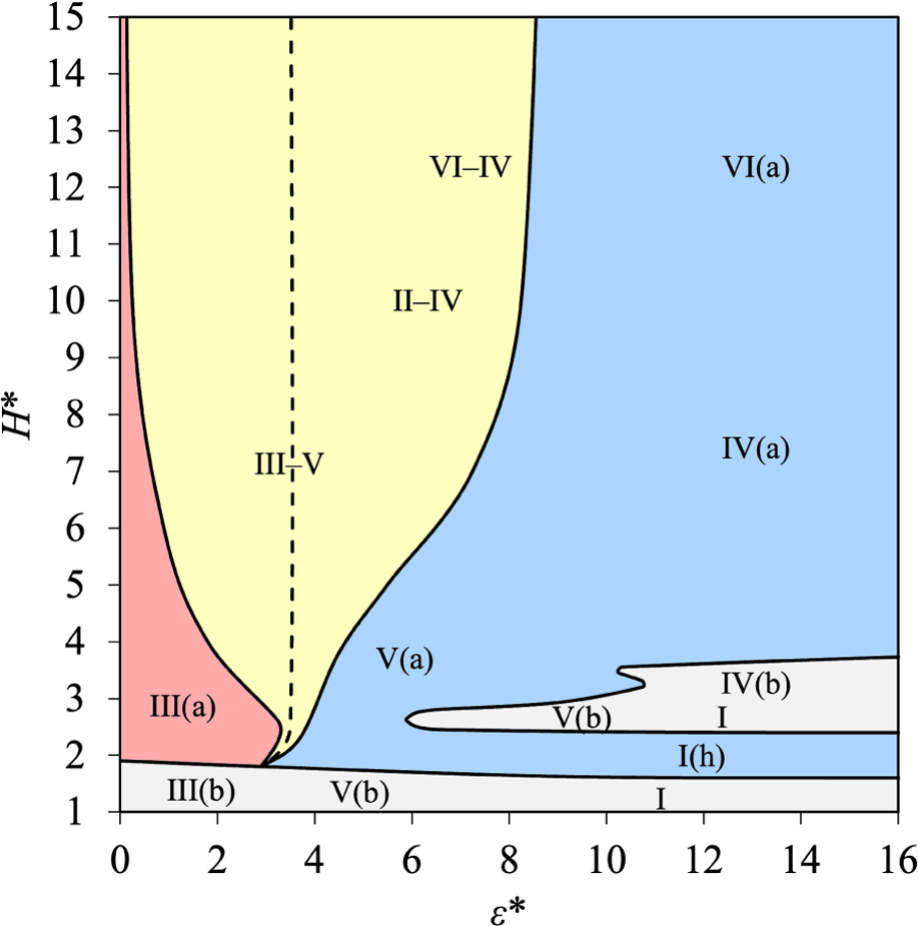
Phase diagram at constant temperature, T* = 0.70, expressed in terms of the pore size and the fluid–wall interaction energy (H*–ε* diagram). The curves and colored regions have the same meaning as in Figure 10.

In Figure 12, we present the T*–H* diagram obtained for ε* = 8.0, i.e., for a constant fluid–wall interaction energy. Only Topologies A–D can be seen, as could be expected from the results shown in Figures 10 and 12. For T* ≥ 1.3, i.e., for supercritical temperatures, Topology A is found with Type I(s) for micropores and Type II(s) for meso- and macropores. Types I and IV­(b) can be seen for Topology B as ε* = 8.0 corresponds to strong fluid–wall interactions. The range of temperature for which Type IV­(b) is found decreases for larger pore sizes. The consequence of this is that it is easier to find Type IV­(b) for small mesopores (as mentioned in IUPAC 2015 ^1^) than for macropores, for which the range of temperature can be small (close to the critical temperature *T*_c_ of the bulk fluid). Capillary condensation (with Topology C) is found with Types IV­(a), VI­(a), and I­(h). It is interesting to see that capillary condensation is found for any pore size if the temperature is low enough (even if the size of the hysteresis cycle is very small for Type I­(h) and is commonly neglected). The effect of confinement on the microscopic structure and the creation of adsorption layers in the smallest pores, mentioned in Figure 11, is clearly visible in Figure 12 by following the curve that separates Topologies B and C. Other effects of pore size can be seen for the transition from Topology C to Topology D, with the transitions from Type VI­(a) to Type VI–IV at low temperature and from Type IV­(a) to Type II–IV at higher temperature, by considering larger pores.

**Figure 12:**
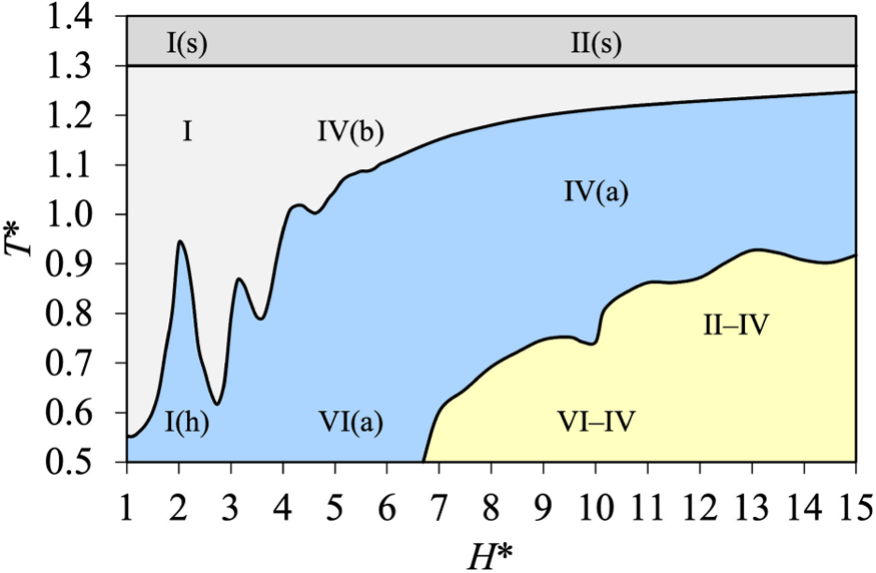
Phase diagram at constant fluid–wall interaction energy, ε* = 8.0, expressed in terms of the pore size and the temperature (H*–T* diagram). The lines and colored regions have the same meaning as in Figure 10.

## 4 Conclusions

In our current work, we revisit the mechanisms of adsorption/desorption and intrusion of gases, liquids, and supercritical fluids into porous substrates using classical DFT. Such an approach overcomes the limitations inherent in the multiplicity and specificity of the existing empirical methods traditionally used to analyze isotherms of physisorption. We are now in a position to answer the questions listed in the Introduction section.

(1) For the physisorption of gases, can we obtain all of the types of isotherms of the 2015 IUPAC classification^1^ with classical DFT, and can we find new types of isotherms?

All types of isotherms defined in IUPAC 2015 can be found with classical DFT. We detail the thermodynamic conditions for which each type can be found, and we present them as diagrams, according to a wide range of temperatures, pore sizes, and intensities of fluid–wall interactions. We suggest renaming Type V of IUPAC 2015 as Type V­(a) as we define a new type, denoted as Type V­(b). Isotherms of Type V­(b) can be found for mesopores with weak fluid–wall interactions (as for isotherms of Type V­(a)), but they are reversible (in contrast to isotherms of Type V­(a)). A system that exhibits an isotherm of Type V­(a) at a given temperature can exhibit an isotherm of Type V­(b) at a higher temperature. This means that a clear analogy can be seen between Types IV­(a) and IV­(b) on one hand and Types V­(a) and V­(b) on the other hand.

(2) What possible topologies and new types of physisorption isotherms are observed by considering gases, liquids, and supercritical fluids within the same theoretical framework?

Five topologies of physisorption isotherms are found by considering the possible VLE of the bulk fluid and the possible irreversibility of the isotherm for the confined fluid. Hysteresis cycles correspond to capillary condensation for gas adsorption (Topology C) and capillary evaporation for liquid intrusion (Topology E), but this may also indicate delayed condensation and evaporation compared with the VLE of the bulk fluid on the same isotherm (Topology D).

New types of isotherms are defined from Types II and III of IUPAC 2015 by considering the saturation of the pore (even for macropores), which can occur only at high pressure when the bulk is a liquid. Type III of IUPAC 2015 can correspond to Types III–V, III­(a), or III­(b) of our new classification. We denote Type II of IUPAC 2015 as Type II–IV to highlight that the full isotherm associated with it is not expected to be reversible. We also propose hypothetical Types II­(a) and II­(b), but these are not found for the systems considered in our current work. Our new classification is based on all of the fundamental mechanisms described within the DFT framework for confined gases, liquids, and supercritical fluids.

The topologies and the types of our classification can be presented in a consistent manner on diagrams (considering the temperature, pore size, and intensity of the fluid–wall interactions), which can guide new analyses of porous materials and new techniques of characterization.

(3) Can we revisit the mechanisms of adsorption/desorption and intrusion into macropores, and how do these mechanisms affect the reversibility of the isotherms?

A large hysteresis cycle can be found on the isotherms of physisorption for macropores. This cycle can only be found for a wide range of the bulk pressure, spread below and above the bulk vapor pressure *P* ^0^ (otherwise, the isotherm is not fully explored and may seem reversible). Such isotherms have Topology D (as defined in Section 2.3) and can correspond to Types III–V, II–IV, or VI–IV, according to the temperature, pore size, and the fluid–wall interaction energy.

(4) What are the topologies of the isotherms of physisorption for fluids confined into micropores?

The most common type of isotherm found for micropores is Type I, i.e., the Langmuir isotherm.^4^ Types V­(b) and III­(b), as defined in our classification, could also be found for micropores for weak and very weak fluid–wall interactions, respectively. However, these isotherms may be found for systems that are not frequently used in applications. Types V­(b) and III­(b) can be ignored most of the time in practice, which explains why Type I is often presented as the only type found for micropores. Surprisingly, we also find that a tiny hysteresis cycle can exist on the isotherms predicted for micropores with strong fluid–wall interactions at very low temperature. We assume that such a hysteresis cycle is too small to be easily found with experiments, such that it is neglected in practice. Nevertheless, we decide to refer to such a behavior as Type I­(h) to emphasize the topological difference with Type I (as Types I­(h) and I correspond to Topologies C and B, respectively). This also means that, in theory, capillary condensation or evaporation can be found for any pore size (i.e., for micropores, mesopores, and macropores) since the temperature is small enough (even for rough surfaces).

(5) What is the effect of temperature on the shape of the physisorption isotherms?

In practice, adsorption and desorption isotherms are often considered at specific temperatures, e.g., 77 K for nitrogen or 87 K for argon. These choices are relevant to the standardization of the techniques used to characterize porous materials. However, a wider range of temperatures must be considered for a given system in order to understand the transitions from a type of isotherm to another type, by changing the temperature. We identified several transitions from low to high temperature: we find Types III–V, III­(a), III­(b), and III(s) for very weak fluid–wall interactions; Types III–V, V­(a), V­(b), and III(s) for weak fluid–wall interactions; Types VI–IV, II–IV, IV­(a), IV­(b), and II(s), or Types VI–IV, VI­(a), IV­(a), IV­(b), and II(s) for mesopores and macropores with strong fluid–wall interactions; and Types I­(h), I, and I(s) for micropores with strong fluid–wall interactions. These transitions are employed to organize our classification in Figures 7 and 8. We also observe that some types can be found for a very small temperature range (e.g., Types IV­(b) and V­(b) for mesopores). Such a sensitivity with temperature may be used to improve the techniques of characterization, e.g., by comparing several isotherms at various temperatures, for a given system.

The proposed classification is not exhaustive. Our work should be extended to complex real fluids (e.g., for molecules with chains and hydrogen bonds) that could be modeled with the SAFT-VR Mie or SAFT-γ Mie EoS. It is well-known that the pore geometry has an effect on the shape of hysteresis cycles,^1^ which means that diverse pore geometries should be included in future studies to extend our results for Topologies C, D, and E. Charges could also be included, in models of both the fluid molecules and activated surfaces. Distinct mechanisms should be revisited, e.g., by considering chemisorption or flexible materials. All of these considerations would most likely lead to different diagrams with temperature, pore size, and fluid–wall energy (and could give rise to the hypothetical Types II­(a) and II­(b)). However, the relations between the topologies obtained for the systems considered in our work must remain for any other systems.

## Data Availability

underlying this article can be accessed on Zenodo at https://zenodo.org/records/18219485 and used under the Creative Commons Attribution license.
